# Conductive-Polymer-Based Double-Network Hydrogels for Wearable Supercapacitors

**DOI:** 10.3390/gels10110688

**Published:** 2024-10-24

**Authors:** Bu Quan, Linjie Du, Zixuan Zhou, Xin Sun, Jadranka Travas-Sejdic, Bicheng Zhu

**Affiliations:** 1 Centre for Innovative Materials for Health, School of Chemical Sciences, The University of Auckland, 23 Symonds Street, Auckland 1010, New Zealand; bqua081@aucklanduni.ac.nz (B.Q.); ldu279@aucklanduni.ac.nz (L.D.); xsun230@aucklanduni.ac.nz (X.S.); j.travas-sejdic@auckland.ac.nz (J.T.-S.); 2 Key Laboratory of Advanced Civil Materials of Ministry of Education, School of Materials Science and Engineering, Tongji University, Shanghai 200070, China; xuanzi_1996@126.com; 3 MacDiarmid Institute for Advanced Materials and Nanotechnology, Victoria University of Wellington, Wellington 6012, New Zealand

**Keywords:** epidermal bioelectronics, flexible supercapacitors, conducting polymer, double-network hydrogels, energy supply, solid-state electrolyte

## Abstract

In the field of contemporary epidermal bioelectronics, there is a demand for energy supplies that are safe, lightweight, flexible and robust. In this work, double-network polymer hydrogels were synthesized by polymerization of 3,4-ethylenedioxythiophene (EDOT) into a poly(vinyl alcohol)/poly(ethylene glycol diacrylate) (PVA/PEGDA) double-network hydrogel matrix. The PEDOT-PVA/PEGDA double-network hydrogel shows both excellent mechanical and electrochemical performance, having a strain up to 498%, electrical conductivity as high as 5 S m^−1^ and specific capacitance of 84.1 ± 3.6 mF cm⁻^2^. After assembling two PEDOT-PVA/PEGDA double-network hydrogel electrodes with the free-standing boron cross-linked PVA/KCl hydrogel electrolyte, the formed supercapacitor device exhibits a specific capacitance of 54.5 mF cm⁻^2^ at 10 mV s^−1^, with an energy density of 4.7 μWh cm^−2^. The device exhibits excellent electrochemical stability with 97.6% capacitance retention after 3000 charging–discharging cycles. In addition, the hydrogel also exhibits great sensitivity to strains and excellent antifouling properties. It was also found that the abovementioned hydrogel can achieve stable signals under both small and large deformations as a flexible sensor. The flexible and antifouling PEDOT-PVA/PEGDA double-network hydrogel-based supercapacitor is a promising power storage device with potential applications in wearable electronics.

## 1. Introduction

The demand for efficient wearable energy storage solutions has surged with the increasing use of epidermal bioelectronics for health monitoring. Research efforts have focused on developing organic, flexible and stretchable energy storage devices to meet the need. Among various energy storage technologies, batteries and supercapacitors emerge as two of the most prominent solutions. Batteries are widely utilized in consumer electronics due to their high energy density [1,2]. However, they can generate heat by potentially causing short circuits, which raises safety concerns [3,4]. In contrast, supercapacitors offer several advantages over batteries, such as rapid charging and discharging, longer cycle life, higher power density and environmental sustainability. As a result, supercapacitors are increasingly used in electronic devices and gaining significant attention [5].

Conducting polymers (CPs) are considered one of the most promising electrode materials for flexible supercapacitor applications due to their high specific capacitance, good conductivity, low cost and ease of fabrication [6]. In recent years, poly(3,4-ethylenedioxythiophene) (PEDOT) has emerged as a leading capacitive material because of its high conductivity, low interface impedance, high charge storage capacity and excellent environmental stability [2]. To meet the flexibility requirements of wearable devices, hydrogel forms of CP-based materials have been studied in order to overcome the rigidity and inflexibility of conducting polymers [1,7]. Polyvinyl alcohol (PVA) has been widely used as a soft matrix to combine effectively with rigid conducting polymers [8]. Lu et al. reported a preparation method for creating a semicrystalline conducting polymer hybrid hydrogel through the supramolecular assembly of PEDOT and PVA for use in wearable supercapacitors [9]. This hydrogel exhibited a high energy density of 24 Wh kg^−1^ and excellent electrochemical stability, retaining 100% of its capacitance after 15,000 cycles. Similarly, Chen et al. designed robust PEDOT-based hydrogels by assembling PEDOT and PVA via dynamic boronated bonds [10]. This supercapacitor achieved an energy density of 15.2 Wh kg^−1^, with a power density of 201.1 W kg^−1^. The device demonstrated impressive durability, with 89% capacitance retention after 1000 charge–discharge cycles and nearly 100% capacitance retention after 1000 mechanical folding cycles at a scan rate of 5 A g^−1^.

It is well known that single polyvinyl alcohol (PVA) hydrogels struggle to meet the mechanical strength requirements for certain applications. Significant efforts have been made to improve the mechanical properties of hydrogels, and constructing double-network (DN) structures is a common and effective approach [11,12]. DN hydrogels consist of two distinctly different polymer networks: the first network is a highly cross-linked polyelectrolyte that acts as a rigid scaffold to maintain its shape, while the second network is a loosely cross-linked flexible neutral polymer that serves as a filler, absorbing external stress [13,14]. As a result, DN hydrogels not only exhibit excellent physical properties like elasticity, high moisture content and superior light transmission, but also address the shortcomings of hydrogels with poor mechanical properties [15]. Kishi et al. synthesized a novel conductive and mechanically tough double-network polymer hydrogel (E-DN gel) by polymerizing 3,4-ethylenedioxythiophene (EDOT) in the presence of a DN gel matrix [16]. The E-DN gel demonstrated exceptional mechanical performance, with a fracture stress of 1.4–2.1 MPa, and exhibited a high conductivity of up to 10^−3^ S cm^−1^. Qin et al. developed a DN hydrogel electrode for all-solid-state supercapacitors by incorporating liquid crystal (LC) clusters and a physically cross-linked PVA network [17]. The resulting hydrogel electrode had an unusually high active material content (25 wt.%), leading to an areal capacitance of 871.4 mF/cm^2^ and areal energy density of 0.14 mWh/cm^2^ at 0.27 mW/cm^2^.

In this work, we developed a flexible supercapacitor device based on a soft, conductive, double-network polymer hydrogel composed of PVA, poly(ethylene glycol) diacrylate (PEG-DA) and 3,4-ethylenedioxythiophene (EDOT). To construct a tough, conductive hydrogel, PVA was used to form crystalline domains through freeze–thaw cycles, serving as the first network. PEGDA was then introduced as the second network, which was cross-linked via covalent bonds. PEDOT was incorporated into the double-network hydrogel to provide both the capacitance and conductivity to the hydrogel. Additionally, the hydrogel exhibited excellent antifouling properties due to the incorporation of PEGDA. The PEDOT-PVA/PEGDA hydrogel demonstrated outstanding mechanical and electrochemical performance, with a strain in the range of 1–498%, electrical conductivity as high as 50 mS cm^−1^, and a specific capacitance of 84.1 ± 3.6 mF cm^−2^. When assembled with a PVA-based hydrogel electrolyte, the resulting supercapacitor exhibited a specific capacitance of 54.5 mF cm^−2^ at 10 mV s^−1^ and an energy density of 4.7 μWh cm^−2^. The hydrogel also displayed excellent electrochemical stability, retaining 97.6% of its capacitance after 3000 charge–discharge cycles. To the best of our knowledge, this is the first PEDOT in a PVA and PEGDA DN hydrogel for a flexible supercapacitor device, facilitating potential applications in wearable sensors and flexible wearable bioelectronics. The PEDOT-PVA/PEGDA DN hydrogel provides excellent mechanical stability, superior electrochemical performance and sensitivity to tensile strain, which offers great potential to be integrated with wearable bioelectronic devices as an energy storage component.

## 2. Results and Discussion

### 2.1. Development of PEDOT-PVA/PEGDA DN Hydrogels for Supercapacitors

**Figure 1:**
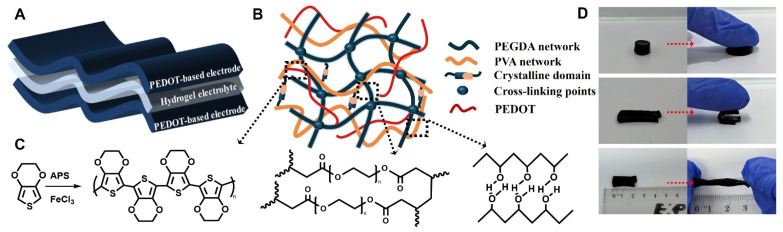
(**A**) The “sandwich” structure of the PEDOT-PVA/PEGDA DN hydrogel-based supercapacitor. (**B**) The double-network structure of the PEDOT-PVA/PEGDA DN hydrogels. (**C**) Chemical polymerization of EDOT and chemical structures of PEGDA and PVA. (**D**) Optical photographs showing PEDOT-PVA/PEGDA DN hydrogels under compression, bending, stretching, twisting and torsional stretching.

The schematic of the PEDOT-PVA/PEGDA DN hydrogel supercapacitor and the structural representation of the PEDOT-PVA/PEGDA hydrogel are shown in Figure 1A,B, respectively. In the chemical structure of the PEDOT-PVA/PEGDA DN hydrogel, physical crosslinking occurs via intermolecular hydrogen bonding and chain entanglement between PVA chains, forming a network that is further stabilized by PVA crystalline domains [18]. During the freeze–thaw process, PVA molecules in the mixed solution were frozen at −20 °C, causing the molecular chains to align and connect in an orderly manner, leading to the formation of microcrystalline regions that act as physical cross-linking points for the PVA molecular chains. Upon thawing, several PVA segments were activated and rearranged during subsequent freezing cycles, thereby improving the microcrystalline structure and forming the hydrogel [19]. The other chemical crosslinked network of DN was formed via the chemical polymerization of PEGDA in the presence of the oxidant of APS [20]. The EDOT was polymerized in the presence of chemical oxidants of FeCl₃ and APS, forming PEDOT, which is interwoven within the PVA/PEGDA DN structure (Figure 1C). A binary oxidant system consisting of FeCl₃ and ammonium persulfate (APS) was employed for chemical polymerization. Although APS has low solubility in DMSO, it offers a high polymerization rate. Additionally, the Cl^−^ ions from FeCl_3_ acted as dopant ions for the conducting polymer, thereby enhancing its conductivity [21]. A small amount of PSS was added to enhance the uniform dispersion of EDOT monomers within the PVA/PEGDA DN structure [22]. The PEDOT-PVA/PEGDA DN hydrogel demonstrated satisfactory mechanical strength, allowing it to be repeatedly compressed, bent, stretched, twisted and torsionally stretched without compromising its integrity (Figure 1D). It had a high water content of 87%, with the kinetics of water loss shown in Figure S1A.

**Figure 2:**
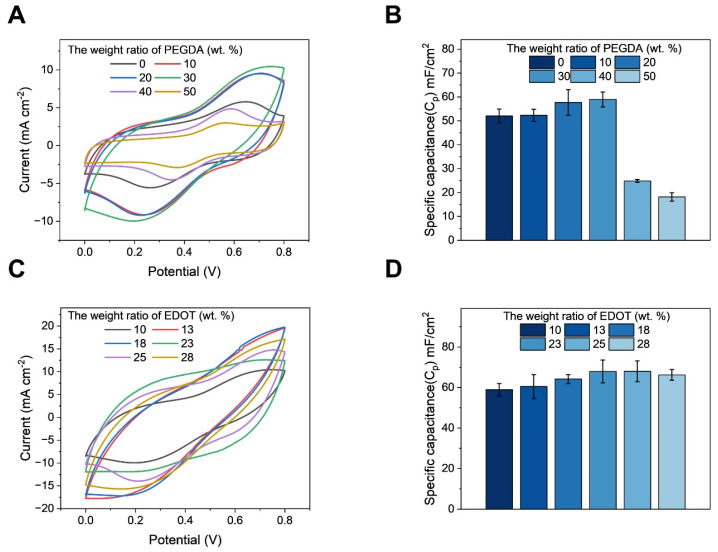
(**A**) CVs at different scan rates and (**B**) areal specific capacitances of PEDOT-PVA/PEGDA DN hydrogel electrodes with PEGDA content from 0 wt.% to 50 wt.% in 1 M KCl solution at a scan rate of 100 mV s^−1^. (**C**) CVs at different scan rates and (**D**) areal specific capacitances of PEDOT−PVA/PEGDA DN hydrogel electrodes with EDOT content from 10 wt.% to 28 wt.% in 1 M KCl solution at a scan rate of 100 mV s^−1^.

To find the optimum capacitance of the PEDOT-PVA/PEGDA DN hydrogel, the composition of the PEDOT-PVA/PEGDA DN hydrogel electrode was optimized by investigating the effect of the contents of both PEGDA and EDOT during the preparation of the PEDOT-PVA/PEGDA DN hydrogel. Figure 2A shows CV curves of the electrodes obtained with different PEGDA weight ratios in the total solution, and their specific capacitances were calculated and presented in Figure 2B. When 30 wt.% of PEGDA in the total solution was added, the PEDOT-PVA/PEGDA DN hydrogel exhibited an optimum capacitance of 58.9 ± 3.1 mF cm^−2^ at a scan rate of 100 mV s^−1^. The appropriate weight ratio of PEGDA allowed PEDOT to be more evenly distributed throughout the double networks, creating a more efficient charge transport pathway. This uniform distribution in turn could enhance both conductivity and capacitance [23]. However, when the weight ratio of PEGDA content in the total solution was increased to 40 wt.%, the network structure of PEGDA may become overly dense, restricting ion migration and leading to a decrease in capacitance. Therefore, 30 wt.% in the total solution was utilized as the optimum amount of PEGDA for further study.

The weight ratio of EDOT in the total solution was then optimized, as shown in Figure 2C. When more than 23 wt.% of EDOT in the total solution was added, the capacitance reached an optimum value of 67.9 ± 5.6 mF cm^−2^ and then saturated. There was almost no change in capacitance when comparing samples prepared from 23 wt.% and 28 wt.% of EDOT (Figure 2D). Therefore, the optimal composition of PEGDA and EDOT for preparing the hydrogel electrode were determined as 30 wt.% and 23 wt.% in the total solution, respectively.

### 2.2. Characterizations of the PEDOT-PVA/PEGDA DN Hydrogel

**Figure 3:**
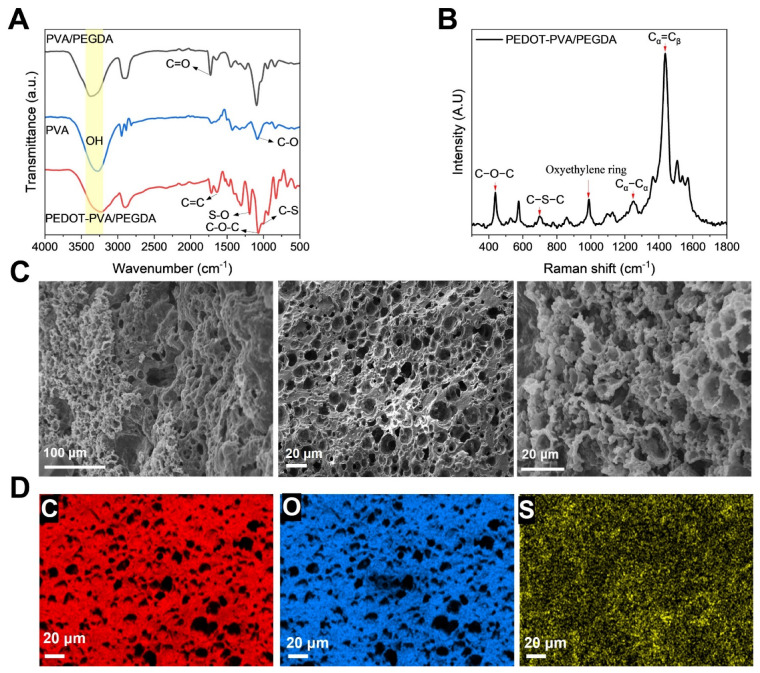
(**A**) FTIR spectra of the dried PVA, PVA/PEGDA and PEDOT-PVA/PEGDA DN hydrogels. (**B**) Raman spectra of dried PEDOT-PVA/PEGDA DN hydrogel. (**C**) SEM morphologies of the fracture surfaces of the freeze-dried PEDOT-PVA/PEGDA DN hydrogels. (**D**) EDX elemental mapping images of freeze-dried PEDOT-PVA/PEGDA DN hydrogel: C element, O element and S element.

The chemical structure of the PEDOT-PVA/PEGDA DN hydrogel was investigated using FT-IR and Raman spectroscopy; the spectra are shown in Figure 3A,B. In the FT-IR spectra of EDOT-PVA/PEGDA, peaks at 980 cm^−1^, 1050 cm^−1^, 1293 cm^−1^ and 1635 cm^−1^ were observed, which correspond to C–S stretching, C–O–C stretching, S–O stretching and C=C stretching of PEDOT, indicating that PEDOT is present, as expected, in the developed PEDOT-PVA/PEGDA DN hydrogel. In the FT-IR spectra of PVA/PEGDA and PVA, the peak at 1150 cm^−1^ is associated with the stretching vibration of C-O of the remaining nonhydrolyzed vinyl acetate group in the PVA, and the peak at 1720 cm^−1^ corresponds to the C=O stretching of PEGDA [24]. These findings confirm the coexistence of PEDOT, PEGDA and PVA within the DN hydrogel. After the hydrogel formation, the characteristic peak of PVA at 3288 cm^−1^ was shifted to the red, reflecting the hydrogen bonding that occurs after cross-linking.

The Raman spectrum of the PEDOT-PVA/PEGDA DN hydrogel (Figure 3B) reveals characteristic peaks at 439 cm^−1^ and 698 cm^−1^, corresponding to C–O–C and C–S–C deformations, respectively. Peaks at 991 cm^−1^ and 1251 cm^−1^ are assigned to oxyethylene ring deformation and Cα–Cα inter-ring stretching. A prominent peak at 1435 cm^−1^ is attributed to symmetric C_α_=C_β_ stretching, reflecting a high degree of conjugation within the PEDOT chains. These findings suggest that the incorporation of PVA and PEGDA has minimal impact on the polymerization of EDOT and the resulting PEDOT chain architecture. In comparison, the Raman spectrum of commercial PEDOT displays a peak at ~1441 cm^−1^, indicative of a shorter conjugation length relative to the PEDOT in the PEDOT-PVA/PEGDA DN hydrogel [25].

The morphologies of the fracture surfaces of the prepared hydrogels after freeze-drying were examined using SEM (Figure 3C). The hydrogels exhibited a highly porous structure with an average pore size of approximately 4 μm. Such a porous network structure was discussed by Yang et al. to be advantageous for facilitating the transport of electrolyte ions within the DN structure [26]. Figure 3D presented the EDX elemental mapping of the freeze-dried PEDOT-PVA/PEGDA DN hydrogel, confirming the presence of C, O and S in the PEDOT-PVA/PEGDA DN hydrogels and the uniform distribution of these elements within the hydrogel structure. The elemental analysis (Figure S2) showed freeze-dried PEDOT-PVA/PEGDA DN hydrogel had the composition of 80.1% of C, 17.2% of O, 0.4% of S, 1.1% of Fe and 1.3% of Cl. The presence of Fe and Cl was attributed to residual FeCl_3_ after washing. These findings confirmed that all components were well-mixed and PEDOT was successfully introduced and uniformly distributed within the DN hydrogel matrix.

Due to the presence of PEG groups in the PEDOT-PVA/PEGDA DN hydrogel, the PEDOT-PVA/PEGDA DN hydrogel provided excellent antifouling properties. To test its antifouling performance, the EIS of hydrogel electrodes, before and after 24 h immersion in 10 wt.% BSA solution, was measured in 5 mM Fe[(CN)_6_]^3−/4−^ PBS solution (Figure S1B). The negligible changes in EIS results indicate no adhesion of BSA on the surface. As shown in Figure S3C, the PEDOT-PVA/PEGDA DN hydrogel also exhibited high hydrophilicity according to the contact angle profile of water on its surface of about 20°. Therefore, it is expected to be used in epidermal electronic devices.

### 2.3. Electrochemical Performance of PEDOT-PVA/PEGDA DN Hydrogel as Supercapacitor Electrode

**Figure 4:**
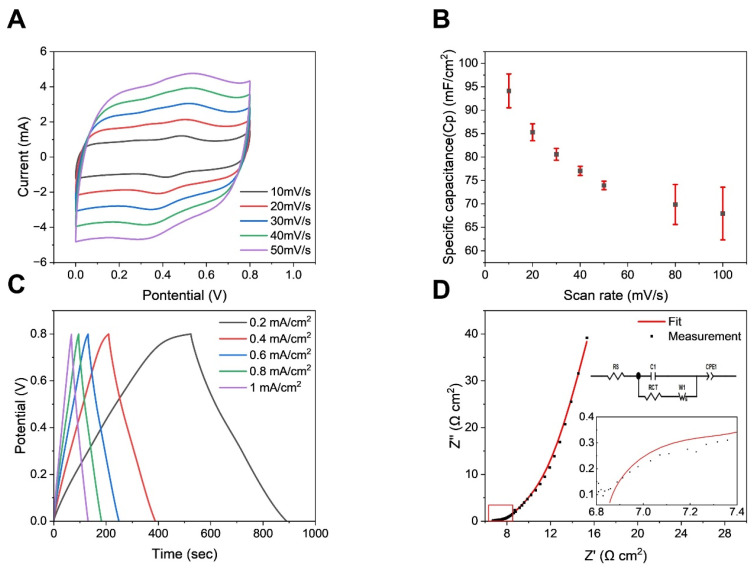
Characterization of PEDOT-PVA/PEGDA DN hydrogel as supercapacitor electrode material. (**A**) CV curves at various scan rates in the potential window of 0–0.8 V. (**B**) Specific capacitance of the PEDOT-PVA/PEGDA DN hydrogel electrode at different scan rates. The error bars represent a standard deviation from 3 measurements. (**C**) GCD curves at a current density from 0.2 mA·cm^−2^ to 1 mA cm^−2^ in a potential window of 0–0.8 V. (**D**) Nyquist plot of PEDOT-PVA/PEGDA DN hydrogel in the frequency range of 0.1–100 kHz.

To evaluate the electrochemical properties of the PEDOT-PVA/PEGDA DN hydrogel as a supercapacitor electrode, PEDOT-PVA/PEGDA DN hydrogel was prepared onto a treated, but still slightly hydrophobic, carbon cloth (Figure S3). PEDOT-PVA/PEGDA DN hydrogel electrodes, prepared under the optimum conditions, were utilized for electrochemical testing. As shown in Figure 4A, the CV curves at different scan rates, between 10 mV s^−1^ and 50 mV s^−1^, exhibited symmetrical shapes with an oxidation peak at 0.5 V and a reduction peak at 0.4 V, indicating the redox property of PEDOT and excellent capacitive behavior of the PEDOT-PVA/PEGDA DN hydrogel electrode. For comparison, a CV measurement of bare CC was also conducted, showing negligible capacitance from bare CC (Figure S4). The specific capacitance calculated from the CV data (Figure 4B) reached a high value of 94.1 ± 3.6 mF cm^−2^ at a scan rate of 10 mV/s. The symmetric charge–discharge curves (Figure 4C) further demonstrate the excellent capacitive characteristics of the PEDOT-PVA/PEGDA DN hydrogel electrode. The GCD curves retained triangular shapes, also confirming the outstanding capacitive properties. To investigate the conductivity mechanism in more detail, the Nyquist plot of the hydrogel (Figure 4D) was obtained by measuring EIS and fitted the spectra to the reported equivalent circuit model shown in the Figure 4D inset [27]. The plot displayed a nearly vertical line in the low-frequency region, indicating typical capacitive behavior for PEDOT-PVA/PEGDA DN hydrogel electrode [28]. In the high-frequency region, a small semicircle appeared, which reflected the fast ion- and charge-transfer kinetics at the PEDOT-PVA/PEGDA DN hydrogel–electrolyte interface [29], where a low equivalent series resistance (R_s_*)* of 6.8 Ω cm^2^ and a low charge-transfer resistance (R_CT_) of 0.6 Ω cm^2^ were obtained. These low resistances were attributed to the high electronic conductivity introduced by PEDOT, where polarons and bipolarons, as charge carriers, are generated during PEDOT polymerization [30]. Moreover, the fitted Warburg impedance (Z_W_) was as low as 0.1 Ω, indicating that the PEDOT-PVA/PEGDA DN hydrogel also exhibited excellent ionic conductivity, which arose from the movement of ions in the DN through the highly hydrated microporous structure [31]. These findings suggested that the PE-DOT-PVA/PEGDA DN hydrogel functioned as a charge carrier with both electronic and ionic conductivity. The fitted parameters from the Nyquist plot and the equivalent circuit model are listed in Table S1.

### 2.4. Mechanical Performance of PEDOT-PVA/PEGDA DN Hydrogel

**Figure 5:**
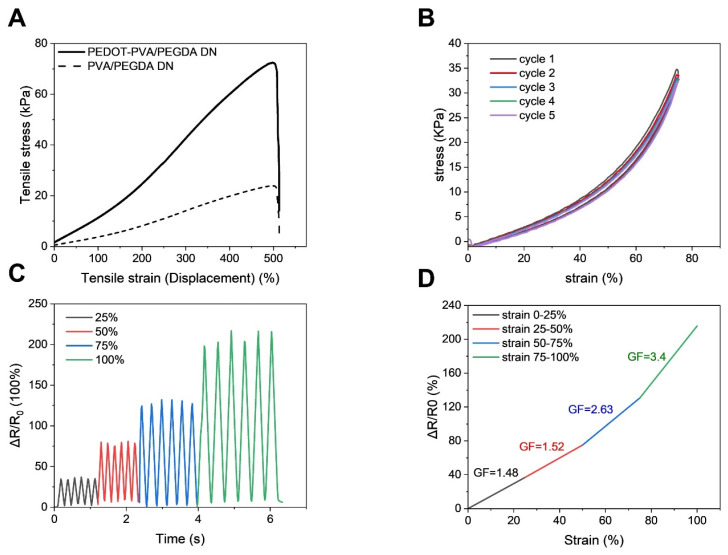
(**A**) Tensile stress–strain curves of PEDOT-PVA/PEGDA DN hydrogel and PVA/PEGDA DN hydrogel. (**B**) Cyclic tensile test of PEDOT-PVA/PEGDA DN hydrogel up to 75% strain. (**C**) The change in the relative resistance (∆*R/R*_0_) of PEDOT-PVA/PEGDA DN hydrogel at different strains (25%, 50%, 75%, 100%). (**D**) *GF* of PEDOT-PVA/PEGDA DN hydrogel at different tensile strain stages.

The highly porous double-network structure provided the hydrogel with excellent mechanical properties. As shown in Figure 5A, the PEDOT-PVA/PEGDA DN hydrogel exhibited a maximum tensile strength of 72 kPa and a Young’s modulus of 15 kPa, with an elongation at break of 498%, while PVA/PEGDA DN hydrogel presented a maximum tensile strength of 23 kPa and a Young’s modulus of 5 kPa. The Young’s modulus of the PEDOT-PVA/PEGDA DN hydrogel was approximately three times that of the PVA/PEGDA DN hydrogel. In contrast, their fracture strain values were nearly identical. The presence of PEDOT within the DN hydrogel matrix appears to have minimal impact on the fracture process [32]. The stress–strain curve in Figure 5A demonstrated that the addition of PEDOT resulted in an enhancement of the mechanical strength of the DN hydrogel. The PEDOT-PVA/PEGDA DN hydrogel had a relatively low elastic modulus, which was consistent with the properties observed in similar hydrogel materials reported in the literature [33]. The conductivity of the hydrogel, calculated based on its resistance and dimensions, was 5.2 S m^−1^, measured using the setup shown in Figure S5. The high conductivity and porous structure could provide more electroactive sites for charge storage, facilitating the transport of charges and electrolyte ions.

A series of cyclic tensile tests were carried out to investigate the energy dissipation mechanism of PEDOT-PVA/PEGDA DN hydrogels. When five consecutive loading–unloading tests were performed up to 75% strain (Figure 5B), hysteresis losses were calculated as less than 10% from the area of the hysteresis loops, indicating that the cross-linked structure of the double network provided the hydrogel with an effective energy dissipation capability. As shown in Figure S6A, when the maximum strains increased from 75% to 100% in the cyclic tensile test, the hysteresis loss increased to 18% (Figure S6B), indicating that more of the crosslinked network was disrupted to dissipate the energy. Compared with other reported literature on hydrogels [34,35,36], PEDOT-PVA/PEGDA DN hydrogel provides superior flexibility and fatigue resistance. Figure 5C showed more stable sawtooth waveforms at small strains (25%, 50%) compared to larger strains (75%, 100%) during stretching. The relative resistance (∆*R/R*_0_), calculated from normalized resistance change (∆*R*) and the original resistance (*R*_0_), was found to increase with higher strain. The ∆*R/R*_0_ reached 38%, 78%, 131% and 216% at 25%, 50%, 75% and 100% strain and went back to 0 when released to the original length. Such an increase in Δ*R/R*_0_ under stretching is mainly attributed to the deformation of ion channels and the longer ion migration paths under stretching, leading to a decrease in ion migration rate and an increase in resistance [37]. As shown in Figure 5D, the change rate of Δ*R/R*_0_ of the hydrogel increased with the tensile strain applied. The sensitivity of the PEDOT-PVA/PEGDA DN hydrogel was evaluated from the gauge factor (*GF*) [38]. *GF* can be determined from the slope of the linear curves at different strains in the tensile test as shown in Equation (1):

(1) $$GF=\frac{\Delta R∕R_{0}}{\epsilon }$$

where *R*_0_ represents the initial resistance under no strain, Δ*R* is the change in resistance and *ϵ* denotes the applied strain (Figure 5D). The relative resistance changed with increasing strain and exhibited a linear behavior within several regions of tensile strain, with the following linear fits for each region: (0–25% strain) Δ*R/R*_0_ = 1.5 × strain (%) −2.5 (R^2^ > 0.99), (25–50% strain) Δ*R/R*_0_ = 1.5 × strain (%) −1.1 (R^2^ > 0.99), (50–75% strain) Δ*R/R*_0_ = 2.2 × strain (%) −36.1 (R^2^ > 0.99) and (75–100% strain) Δ*R/R*_0_ = 3.4 × strain (%) −124.6 (R^2^ > 0.99), respectively. In addition, the relative resistance of the hydrogel exhibited three distinct regions with different slopes: in the low strain region of 0–50%, the *GF* was approximately 1.5, and increased to 2.6 in the medium strain region of 50–75%, reaching 3.4 in the high strain region of 75–100%. The *GF* value increased as the applied strain increased. Such a change in sensitivity is likely due to the hydrogel undergoing an increase in deformation during stretching, resulting in a change in the ion conduction path. Consequently, this leads to a slower migration of ions within the hydrogel [38,39]. Therefore, the PEDOT-PVA/PEGDA DN hydrogel demonstrated the ability to be utilized as flexible strain sensor with stable signals under both small and large deformations.

### 2.5. Electrochemical Performance of PEDOT-PVA/PEGDA DN Hydrogel-Based Flexible Supercapacitor

**Figure 6:**
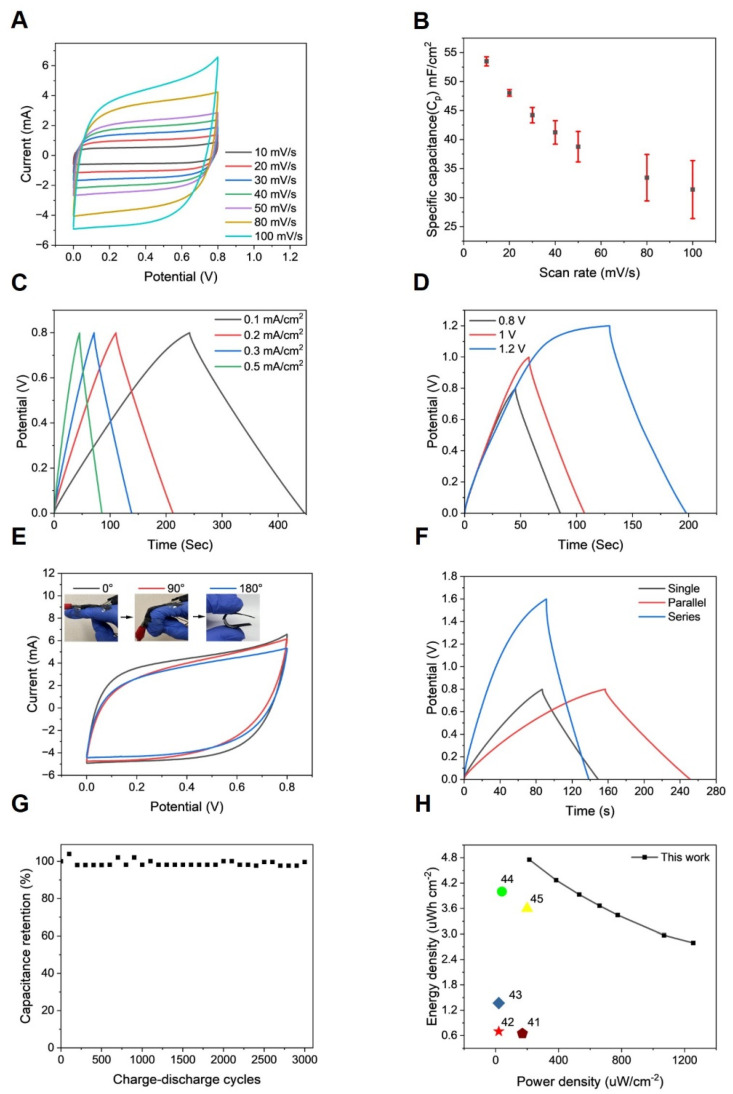
Characterizations of PEDOT-PVA/PEGDA DN hydrogel-based supercapacitor device. (**A**) CV plots at different scan rates in the voltage window of 0–0.8 V. (**B**) Specific capacitance plot of PEDOT-PVA/PEGDA DN hydrogel-based supercapacitors at different scan rates. The error bars represent a standard deviation from 3 measurements. (**C**) GCD curves at a current density from 0.1 mA·cm^−2^ to 0.5 mA cm^−2^ in voltage windows of 0–0.8 V. (**D**) GCD curves at a current density of 1 mA cm^−2^ in various voltage windows. (**E**) CV plots of the PEDOT-PVA/PEGDA DN hydrogel-based supercapacitor at different bending angles. (**F**) GCD curves of a single PEDOT-PVA/PEGDA DN hydrogel-based supercapacitor, two PEDOT-PVA/PEGDA DN hydrogel-based supercapacitors connected in parallel and two PEDOT-PVA/PEGDA DN hydrogel-based supercapacitors connected in series. (**G**) Capacitance retention (%) during GCD cyclic test at a current density of 3 mA cm^−2^. (**H**) Ragone plots of comparison with various PEDOT-based supercapacitors.

In order to assess the capabilities of the PEDOT-PVA/PEGDA DN hydrogel electrode for HSC applications, a sandwiched device was constructed using two PEDOT-PVA/PEGDA DN hydrogel electrodes and a PVA/KCl hydrogel-based electrolyte, as illustrated schematically in Figure 1A. The CV curves of PEDOT-PVA/PEGDA DN hydrogel-based flexible supercapacitor at scan rates ranging from 10 to 100 mV s^−1^ exhibited quasi-rectangular shapes (Figure 6A), showing excellent charge storage capability. As the scan rate increased from 10 to 100 mV s^–1^, the areal capacitance decreased from 54.5 to 31.4 mF cm^–2^ (Figure 6B). The GCD curves (Figure 6C) maintained triangular shapes at various current densities, consistent with the CV results. GCD curves across different potential windows from 0.8 to 1.2 V (Figure 6D) were examined, which also displayed ideal triangular charge–discharge patterns. In order to meet the particular energy and power requirements of practical applications, multiple supercapacitors were configured in series or parallel for the purpose of performance assessment (Figure 6F). In a parallel configuration, the discharge time was twice that of a single supercapacitor at the same current density. Conversely, in a series setup, the devices demonstrated a 1.6 V charge–discharge voltage window, maintaining equal discharge times when compared to the single device. As demonstrated in Figure 6G, the PEDOT-PVA/PEGDA DN hydrogel supercapacitor showed excellent electrochemical stability, with 97.6% capacitance retention after 3000 GCD cycles within a potential window between 0 and 0.8 V at a current density of 3 mA cm^−2^. Conductive-polymer-based supercapacitors often experience volume expansion and contraction during charge–discharge cycles, limiting their cycling stability [6]. The excellent electrochemical stability of the PEDOT-PVA/PEGDA DN hydrogel supercapacitor is attributed to the introduction of the double-network structure, which enhances the mechanical properties and resistance to interference of the hydrogel. This structure supports and protects the PEDOT framework during expansion and contraction [40]. Moreover, mechanical deformation did not negatively affect electrochemical performance of the PEDOT-PVA/PEGDA DN hydrogel supercapacitor. As shown in Figure 6E, the PEDOT-PVA/PEGDA DN hydrogel supercapacitor adhered to the knuckle of a finger and the CV curves were recorded for the PEDOT-PVA/PEGDA supercapacitor device under 0°, 90° and 180° of bending degree, where 0° and 90° were measured on the finger. Almost no change in the curves was observed, highlighting the device’s excellent mechanical flexibility, stability and excellent application potential as an epidermal electronic energy storage device. Figure 6H presented the Ragone plot, comparing the electrochemical performance of the PEDOT-PVA/PEGDA DN hydrogel supercapacitor with other PEDOT-based supercapacitors in terms of energy density and power density [41,42,43,44,45]. Compared to previously reported PEDOT-based flexible supercapacitors, listed in Table S2, the supercapacitor prepared in this study demonstrated better energy density and power density, which could result from the porous DN structure that allows the conductive agent PEDOT to be more uniformly dispersed in the hydrogel, facilitating the transport of electrolyte ions and improving the conductivity and capacitance of the hydrogel electrodes.

## 3. Conclusions

We fabricated a flexible PEDOT-based supercapacitor using double-network hydrogel as electrodes. The PEDOT-PVA/PEGDA DN hydrogel electrodes have good properties as flexible strain sensors with stable signals under both small and large deformations. The device demonstrates excellent capacitance, high energy density and power density, remarkable flexibility, great sensitivity and anti-fouling properties. The areal capacitance, energy density and power density reach 54.5 mF cm^–2^, 4.7 μWh cm^–2^ and 213.9 μW cm^–2^, respectively. The high performance is attributed to the uniform distribution of the conductive polymer PEDOT within the porous double-network hydrogel structure, enhancing both capacitance and conductivity through redox reactions. As a result, this supercapacitor holds great potential as a suitable energy storage device for flexible, wearable and epidermal bioelectronics. The excellent electrochemical and mechanical properties exhibited by the prepared PEDOT-PVA/PEGDA DN hydrogels, make them promising candidates for a broad range of bioelectronics applications like flexible energy supplies for wearable devices and flexible electronics, as well as flexible strain sensors for monitoring human movements by detecting changes in the hydrogel’s resistance under stretching.

## 4. Materials and Methods

### 4.1. Materials

The 3,4-Ethylenedioxythiophene (EDOT), ammonium persulfate (APS), iron (III) chloride hexahydrate (FeCl_3_ 6H_2_O), polyethene glycol diacrylate (PEGDA), potassium chloride (KCl) and poly(4-styrenesulfonic acid) solution (PSS) (18 wt.%) were analytical grade and purchased from Sigma-Aldrich (NZ, St. Louis, MO, USA). Poly(vinyl alcohol) (PVA) was purchased from Ajax Finechem Pty Ltd (NZ, New South Wales, Australia). Carbon cloth (CC, ELAT LT 2400W—40 × 40) was purchased from Fullcell (Danbury, CN, USA). Bovine serum albumin (BSA) (lyophilized powder, standard grade pH 7.0) was purchased from pH Scientific (NZ, Auckland, New Zealand).

### 4.2. Preparation of PEDOT-PVA/PEGDA DN Hydrogel

A total of 1 g of PVA was dissolved in 9 g of a mixture of DMSO/water (1:1). After the full dissolution of the PVA, 0.3 g of PEGDA solution was added at 50 °C under stirring. A volume of 20 μL PSS and 1.6 mmol of EDOT were added to the mixture and stirred well at 50 °C. Then, 0.16 mmol of APS was dissolved in 250 μL of deionized (DI) water (Milli-Q, 18.2 MΩ·cm, 25 °C) and iron (III) chloride hexahydrate, FeCl_3_ 6H_2_O; 1.6 mmol in 200 μL of DI water was added to the solution as the oxidant. The color of the mixture solution changed immediately to dark blue as the chemical polymerization of EDOT took place. The reaction was kept at 50 °C for 24 h until completion. Gelation occurred when the mixture cooled down to room temperature. Two freeze–thaw cycles were conducted, facilitating the formation of crystalline domains of PVA. PEDOT-PVA/PEGDA DN hydrogel samples were immersed in DI water for 24 h.

In addition, the composition of PEDOT-PVA/PEGDA DN hydrogel electrodes was investigated by optimizing the ratios of each component, as listed in Table S3. The necessity of DN and the composition of PEGDA were evaluated by measuring and comparing the electrochemical performance of Sample No. 1–6 in Table S3, where the composition of PVA and EDOT were kept constant and various w% of PEGDA were added. To obtain the optimum capacitance of PEDOT-PVA/PEGDA DN hydrogel electrodes, different EDOT concentrations were investigated and their electrochemical performance was measured; see Sample No. 7–12 in Table S3, where the w% of PVA and PEGDA were kept the same with different additions of EDOT.

To prepare the PEDOT-PVA/PEGDA DN hydrogel electrodes, hydrophilic carbon cloth was prepared as the substrate following a previously reported method [10]. The carbon cloth was immersed in nitric acid for 72 h at room temperature, followed by thorough rinsing with water and ethanol. After oxidation, the hydrophilic carbon cloth (CC) was prepared and cut into rectangular pieces with a coating area of 1 cm × 1 cm. The mixture solution for the PEDOT-PVA/PEGDA DN hydrogel was then cast onto the carbon cloth and incubated at 50 °C for 12 h. The resulting PEDOT-PVA/PEGDA DN hydrogel electrodes were thoroughly washed by immersing in DI water for one day.

### 4.3. Assembly of Supercapacitors

The free-standing, cross-linked PVA/KCl hydrogel electrolyte was prepared following a previously reported method [46]. First, 0.15 g of PVA was dissolved in 0.75 mL of deionized water under continuous stirring at 90 °C until a clear solution was obtained. The pH of the PVA solution was then adjusted to 3 using 1 M HCl. Subsequently, 0.25 mL of KCl solution (3 mol L^−1^) and 50 μL of borax solution (0.1 mol L^−1^) were gradually added with stirring. The pH of the resulting PVA/KCl mixture was adjusted to 9 using 25 wt.% ammonia to produce a transparent PVA/KCl hydrogel electrolyte. In order to optimize the generation of air bubbles during the preparation, the whole process was carried out at a low rotational speed with one hour of standing and proper sonication before the addition of ammonia.

Before assembly, the PEDOT-PVA/PEGDA DN hydrogel electrode was immersed in a 3 M KCl solution for 1 h, after which a layer of PVA/KCl gel electrolyte was coated on one side. Two pieces of the PEDOT-PVA/PEGDA DN hydrogel electrodes were then placed together to form a hydrogel-based supercapacitor (HSC). Finally, the HSC was encapsulated with parafilm. 

### 4.4. Characterization of PEDOT-PVA/PEGDA DN Hydrogel

The morphology of the PEDOT-PVA/PEGDA DN hydrogel was analyzed using a scanning electron microscope (SEM, JCM-6000) (Horiba, Kyoto, Japan). The freeze-dried PEDOT-PVA/PEGDA DN hydrogel was sputter-coated with gold before imaging. Fourier-transform infrared (FTIR) spectra were recorded at room temperature using a Bruker VERTEX spectrometer (DE, Billerica, MA, USA), covering a spectrum range of 4000 to 400 cm^−1^. The elemental composition was determined from electron dispersive X-ray (EDX) analysis using Philips XL-30 Environmental Scanning Electron Microscope (ESEM) (Columbus, OH, USA). Raman spectra were obtained with a LabRAM HR Evolution Raman spectrometer (Horiba Japan), using a 532 nm laser at 10% intensity with a 90 s accumulation per scan, repeated ten times. The water content of the PEDOT-PVA/PEGDA DN hydrogel was determined by measuring the sample mass until fully dried. Electrical conductivity was measured by applying various potentials across the PEDOT-PVA/PEGDA DN hydrogel samples and the current was recorded. The mechanical properties of the PEDOT-PVA/PEGDA DN hydrogels were tested on an Instron 5943. For tensile testing, hydrogel samples with a thickness of 2.7 mm were cut into strips (8 mm in width and 50 mm in length) and tested at a strain rate of 10% strain min^−1^. To evaluate the antifouling properties of the PEDOT-PVA/PEGDA DN electrodes, electrochemical impedance spectroscopy (EIS) measurements were conducted before and after the electrodes were immersed in a bovine serum albumin (BSA) solution for 24 h.

### 4.5. Electrochemical Characterization of PEDOT-PVA/PEGDA DN Hydrogel Electrodes

The electrochemical characteristics of the samples were investigated using cyclic voltammetry (CV), electrochemical impedance spectroscopy (EIS) and galvanic current charge–discharge (GCD) measurements recorded by a CHI 660E electrochemical workstation. Electrochemical characterization of the PEDOT-PVA/PEGDA DN hydrogel electrodes was performed in a three-electrode system. The electrolyte used was a 1 M KCl aqueous solution, with a platinum gauze serving as the counter electrode and an Ag/AgCl (vs. 3M KCl) electrode as the reference electrode. Cyclic voltammetry (CV) tests were performed over a voltage range of 0 to 0.8 V vs. the Ag/AgCl (vs. 3M KCl) electrode, with scan rates ranging from 10 to 100 mV s⁻^1^. Electrochemical impedance spectroscopy (EIS) measurements were conducted across a frequency range of 0.1 Hz to 100 kHz at the open-circuit potential, with an amplitude of 10 mV. Galvanostatic charge–discharge (GCD) tests were carried out within a potential range of 0 to 0.8 V at discharge current densities ranging from 0.2 to 1 mA cm^−2^.

The electrochemical performance of the PEDOT-PVA/PEGDA DN hydrogel-based HSC was evaluated using a two-electrode system. CV tests were performed at scan rates ranging from 10 to 100 mV s⁻^1^. Galvanostatic charge–discharge measurements were conducted at current densities from 0.1 to 0.5 mA cm^−2^. Galvanostatic cycling was performed within a voltage range of 0 to 0.8 V at a constant current density of 3 mA cm^−2^ for 3000 cycles. The flexibility of the HSC was assessed by measuring the CV performance under different bending conditions (angles of 90° and 180°), with CV tests recorded at a scan rate of 10 mV s⁻^1^. The surface-specific capacitance (*C_A_*, mF cm^−2^), energy density (*E_A_*, μW h cm^−2^) and power density (*P_A_*, μW cm^−2^) were calculated from CV tests as follows:

(2) $$C_{A}=\frac{2*\int IdV}{v\times A\times \Delta V}$$

(3) $$E_{A}=\frac{C_{A}\times \Delta V^{2}}{2\times 3.6}$$

(4) $$P_{A}=\frac{E_{A}\times 3600}{\Delta t}$$

where ∫Idv is the area of the CV curves, *ν* is the potential scan rate (V s^−1^), *I* is the discharge current (A), Δ*t* is the discharge time (s), Δ*V* is the potential window (V) and *A* is the total area of the electrode (cm^2^).

## Data Availability

The data supporting the reported results can be found in the article and the Supplementary Materials.

## Supplementary Materials

The following supporting information can be downloaded at: https://www.mdpi.com/article/10.3390/gels10110688/s1, Figure S1: (**A**) Plot of the residual weight of the PEDOT-PVA/PEGDA DN hydrogels over time. (**B**) EIS plots of PEDOT-PVA/PEGDA DN hydrogels electrodes before and after being immersed in BSA solution for 24 h; Figure S2: EDX mapping spectrum of the PEDOT-PVA/PEGDA DN hydrogel; Figure S3: Contact angle profile of water on (**A**) an untreated bare carbon cloth surface and (**B**) a bare carbon cloth surface treated with HNO3 (**C**) the PEDOT-PVA/PEGDA DN hydrogel surface; Figure S4 CV curves of PEDOT-PVA/PEGDA DN hydrogel electrode and bare carbon cloth; Figure S5: (**A**) Setup for conductivity measurement. The width, thickness and length of measured hydrogels were 0.7 cm, 0.2 cm and 2 cm. (**B**) i-v curves of PVA/PEGDA DN hydrogel and PEDOT-PVA/PEGDA DN hydrogel. (**B**) Conductivity of the PVA/PEGDA DN hydrogel and PEDOT-PVA/PEGDA DN hydrogel; Figure S6: (**A**) Cyclic tensile test of PEDOT-PVA/PEGDA DN hydrogel up to 75% and 100% strain. (**B**) The tensile hysteresis values of the PEDOT-PVA/PEGDA DN hydrogel at 75%, 100%; Table S1 The parameters fitted from the Nyquist plot; Table S2: The comparison of the PEDOT-PVA/PEGDA DN hydrogel-based supercapacitor with other PEDOT-based flexible supercapacitors; Table S3: Composition of PEDOT-PVA/PEGDA DN hydrogels.

## References

[1] Zhao D., Zhu Y., Cheng W., Xu G., Wang Q., Liu S., Li J., Chen C., Yu H., Hu L. (2020). A Dynamic Gel with Reversible and Tunable Topological Networks and Performances. Matter.

[2] Carlberg J.C., Inganäs O. (1997). Poly(3,4-ethylenedioxythiophene) as Electrode Material in Electrochemical Capacitors. J. Electrochem. Soc..

[3] Hu L., Xu K. (2014). Nonflammable Electrolyte Enhances Battery Safety. Proc. Natl. Acad. Sci. USA.

[4] Choi N.S., Chen Z., Freunberger S.A., Ji X., Sun Y.K., Amine K., Yushin G., Nazar L.F., Cho J., Bruce P.G. (2012). Challenges Facing Lithium Batteries and Electrical Double-Layer Capacitors. Angew. Chem. Int. Ed..

[5] Shao Y., El-Kady M.F., Sun J., Li Y., Zhang Q., Zhu M., Wang H., Dunn B., Kaner R.B. (2018). Design and Mechanisms of Asymmetric Supercapacitors. Chem. Rev..

[6] Shown I., Ganguly A., Chen L.C., Chen K.H. (2015). Conducting Polymer-Based Flexible Supercapacitor. Energy Sci. Eng..

[7] Wang X., Zhou J., Zhu Y., Cheng W., Zhao D., Xu G., Yu H. (2020). Assembly of Silver Nanowires and PEDOT:PSS with Hydrocellulose toward Highly Flexible, Transparent and Conductivity-Stable Conductors. Chem. Eng. J..

[8] Wang K., Zhang X., Li C., Sun X., Meng Q., Ma Y., Wei Z. (2015). Chemically Crosslinked Hydrogel Film Leads to Integrated Flexible Supercapacitors with Superior Performance. Adv. Mater..

[9] Lu H., Li Y., Chen Q., Chen L., Zhang N., Ma M. (2019). Semicrystalline Conductive Hydrogels for High-Energy and Stable Flexible Supercapacitors. ACS Appl. Energy Mater..

[10] Chen Q., Lu H., Chen F., Chen L., Zhang N., Ma M. (2018). Supramolecular Hydrogels for High-Voltage and Neutral-PH Flexible Supercapacitors. ACS Appl. Energy Mater..

[11] Chen Q., Wei D., Chen H., Zhu L., Jiao C., Liu G., Huang L., Yang J., Wang L., Zheng J. (2015). Simultaneous Enhancement of Stiffness and Toughness in Hybrid Double-Network Hydrogels via the First, Physically Linked Network. Macromolecules.

[12] Li L., Wu P., Yu F., Ma J. (2022). Double Network Hydrogels for Energy/Environmental Applications: Challenges and Opportunities. J. Mater. Chem. A Mater..

[13] Yang W., Furukawa H., Gong J.P., Gong J.P., Yang W., Furukawa H. (2008). Highly Extensible Double-Network Gels with Self-Assembling Anisotropic Structure. Adv. Mater..

[14] Huang M., Hou Y., Li Y., Wang D., Zhang L. (2017). High Performances of Dual Network PVA Hydrogel Modified by PVP Using Borax as the Structure-Forming Accelerator. Des. Monomers Polym..

[15] Nonoyama T., Gong J.P. (2021). Tough Double Network Hydrogel and Its Biomedical Applications. Annu. Rev. Chem. Biomol. Eng..

[16] Kishi R., Hiroki K., Tominaga T.F., Sano K.I., Okuzaki H., Martinez J.G., Otero T.F., Osada Y. (2012). Electro-Conductive Double-Network Hydrogels. J. Polym. Sci. B Polym. Phys..

[17] Qin L., Yang G., Li D., Ou K., Zheng H., Fu Q., Sun Y. (2022). High Area Energy Density of All-Solid-State Supercapacitor Based on Double-Network Hydrogel with High Content of Graphene/PANI Fiber. Chem. Eng. J..

[18] Wang Y., Xiang C., Li T., Ma P., Bai H., Xie Y., Chen M., Dong W. (2017). Enhanced Thermal Stability and UV-Shielding Properties of Poly (Vinyl Alcohol) Based on Esculetin. J. Phys. Chem. B.

[19] Li W., Chen W., Ma L., Yang J., Gao M., Wang K., Yu H., Lv R., Fu M. (2023). Robust Double-Network Polyvinyl Alcohol-Polypyrrole Hydrogels as High-Performance Electrodes for Flexible Supercapacitors. J. Colloid. Interface Sci..

[20] Chen X., Dong C., Wei K., Yao Y., Feng Q., Zhang K., Han F., Mak A.F.T., Li B., Bian L. (2018). Supramolecular Hydrogels Cross-Linked by Preassembled Host–Guest PEG Cross-Linkers Resist Excessive, Ultrafast, and Non-Resting Cyclic Compression. NPG Asia Mater..

[21] Lee S., Park C.H. (2018). Conductivity, Superhydrophobicity and Mechanical Properties of Cotton Fabric Treated with Polypyrrole by in-Situ Polymerization Using the Binary Oxidants Ammonium Peroxodisulfate and Ferric Chloride. Text. Res. J..

[22] Wei H., Lei M., Zhang P., Leng J., Zheng Z., Yu Y. (2021). Orthogonal Photochemistry-Assisted Printing of 3D Tough and Stretchable Conductive Hydrogels. Nat. Commun..

[23] Zhang C., Wang M., Jiang C., Zhu P., Sun B., Gao Q., Gao C., Liu R. (2022). Highly Adhesive and Self-Healing γ-PGA/PEDOT:PSS Conductive Hydrogels Enabled by Multiple Hydrogen Bonding for Wearable Electronics. Nano Energy.

[24] Lue S.J., Chen J.Y., Yang J.M. (2007). Crystallinity and Stability of Poly(Vinyl Alcohol)-Fumed Silica Mixed Matrix Membranes. J. Macromol. Sci. Part. B.

[25] Cheng Y., Ren X., Duan L., Gao G. (2020). A Transparent and Adhesive Carboxymethyl Cellulose/Polypyrrole Hydrogel Electrode for Flexible Supercapacitors. J. Mater. Chem. C Mater..

[26] Yang J., Fan Y., Xiong X., Jiang Q., Li P., Jian J., Chen L. (2024). Highly Conductive and Adhesive Wearable Sensors Based on PVA/PAM/SF/PEDOT:PSS Double Network Hydrogels. Appl. Phys. A.

[27] Naderi H.R., Ganjali M.R., Dezfuli A.S. (2018). High-Performance Supercapacitor Based on Reduced Graphene Oxide Decorated with Europium Oxide Nanoparticles. J. Mater. Sci. Mater. Electron..

[28] Lefebvre M., Qi Z., Rana D., Pickup P.G. (1999). Chemical Synthesis, Characterization, and Electrochemical Studies of Poly(3,4-Ethylenedioxythiophene)/Poly(Styrene-4-Sulfonate) Composites. Chem. Mater..

[29] Li Z., Ma G., Ge R., Qin F., Dong X., Meng W., Liu T., Tong J., Jiang F., Zhou Y. (2015). Free-Standing Conducting Polymer Films for High-Performance Energy Devices. Angew. Chem. Int. Ed. Engl..

[30] Shirakawa H., Louis E.J., MacDiarmid A.G., Chiang C.K., Heeger A.J. (1977). Synthesis of Electrically Conducting Organic Polymers: Halogen Derivatives of Polyacetylene, (CH)x. J. Chem. Soc. Chem. Commun..

[31] Gong J.P., Komatsu N., Nitta T., Osada Y. (1997). Electrical Conductance of Polyelectrolyte Gels. J. Phys. Chem. B.

[32] Kishi R., Kubota K., Miura T., Yamaguchi T., Okuzaki H., Osada Y. (2013). Mechanically Tough Double-Network Hydrogels with High Electronic Conductivity. J. Mater. Chem. C Mater..

[33] Nguyen D.M., Wu Y., Nolin A., Lo C.-Y., Guo T., Dhong C., Martin D.C., Kayser L. (2022). V Electronically Conductive Hydrogels by in Situ Polymerization of a Water-Soluble EDOT-Derived Monomer. Adv. Eng. Mater..

[34] Xia S., Zhang Q., Song S., Duan L., Gao G. (2019). Bioinspired Dynamic Cross-Linking Hydrogel Sensors with Skin-like Strain and Pressure Sensing Behaviors. Chem. Mater..

[35] Liu H., Wang X., Cao Y., Yang Y., Yang Y., Gao Y., Ma Z., Wang J., Wang W., Wu D. (2020). Freezing-Tolerant, Highly Sensitive Strain and Pressure Sensors Assembled from Ionic Conductive Hydrogels with Dynamic Cross-Links. ACS Appl. Mater. Interfaces.

[36] Yu X., Zheng Y., Zhang H., Wang Y., Fan X., Liu T. (2021). Fast-Recoverable, Self-Healable, and Adhesive Nanocomposite Hydrogel Consisting of Hybrid Nanoparticles for Ultrasensitive Strain and Pressure Sensing. Chem. Mater..

[37] Yan Y., He C., Zhang L., Dong H., Zhang X. (2023). Freeze-Resistant, Rapidly Polymerizable, Ionic Conductive Hydrogel Induced by Deep Eutectic Solvent (DES) after Lignocellulose Pretreatment for Flexible Sensors. Int. J. Biol. Macromol..

[38] Liu X., Wu Z., Jiang D., Guo N., Wang Y., Ding T., Weng L. (2022). A Highly Stretchable, Sensing Durability, Transparent, and Environmentally Stable Ion Conducting Hydrogel Strain Sensor Built by Interpenetrating Ca2+-SA and Glycerol-PVA Double Physically Cross-Linked Networks. Adv. Compos. Hybrid. Mater..

[39] Lin F., Zhu Y., You Z., Li W., Chen J., Zheng X., Zheng G., Song Z., You X., Xu Y. (2023). Ultrastrong and Tough Urushiol-Based Ionic Conductive Double Network Hydrogels as Flexible Strain Sensors. Polymers.

[40] Liu N., Ma Y., Xu Z., Guo Y., Luo X. (2023). High-Performance Supercapacitor and Antifouling Biosensor Based on Conducting Polyaniline-Hyaluronic Acid Hydrogels. J. Mater. Sci..

[41] Bertana V., Scordo G., Camilli E., Ge L., Zaccagnini P., Lamberti A., Marasso S.L., Scaltrito L. (2023). 3D Printed Supercapacitor Exploiting PEDOT-Based Resin and Polymer Gel Electrolyte. Polymers.

[42] Xu Q., Lu C., Sun S., Zhang K. (2019). Electrochemical Properties of PEDOT: PSS /V2O5 Hybrid Fiber Based Supercapacitors. J. Phys. Chem. Solids.

[43] Shih C.C., Lin Y.C., Gao M., Wu M., Hsieh H.C., Wu N.L., Chen W.C. (2019). A Rapid and Green Method for the Fabrication of Conductive Hydrogels and Their Applications in Stretchable Supercapacitors. J. Power Sources.

[44] Li J., Yan W., Zhang G., Sun R., Ho D. (2021). Natively Stretchable Micro-Supercapacitors Based on a PEDOT:PSS Hydrogel. J. Mater. Chem. C Mater..

[45] Zeng J., Dong L., Sha W., Wei L., Guo X. (2020). Highly Stretchable, Compressible and Arbitrarily Deformable All-Hydrogel Soft Supercapacitors. Chem. Eng. J..

[46] Zhu B., Chan E.W.C., Li S.Y., Sun X., Travas-Sejdic J. (2022). Soft, Flexible and Self-Healable Supramolecular Conducting Polymer-Based Hydrogel Electrodes for Flexible Supercapacitors. J. Mater. Chem. C Mater..

