# Supplementary material for: Conductive-Polymer-Based Double-Network Hydrogels for Wearable Supercapacitors
Source: Gels. 2024 Oct 24;10(11):688. doi: 10.3390/gels10110688 (PMC11594141; doi:10.3390/gels10110688)
Supplement: Supplementary file 1 [file gels-10-00688-s001.zip › gels-3280046-supplementary.pdf]

Supporting information

Conducting Polymer-based Double-Network Hydrogels for Wearable Supercapacitors

Bu Quan[1], Linjie Du[1], Zixuan Zhou[3], Xin Sun[1,2], Jadranka Travas-Sejdic[1,2], Bicheng Zhu[1,2] *

[1] Centre for Innovative Materials for Health, School of Chemical Sciences, The University of Auckland, 23 Symonds Street, Auckland, New Zealand

[2] MacDiarmid Institute for Advanced Materials and Nanotechnology, Victoria University of Wellington, PO Box 600, Wellington, New Zealand

[3] School of Materials Science and Engineering, Key Laboratory of Advanced Civil Materials of Ministry of Education, Tongji University, Shanghai, China

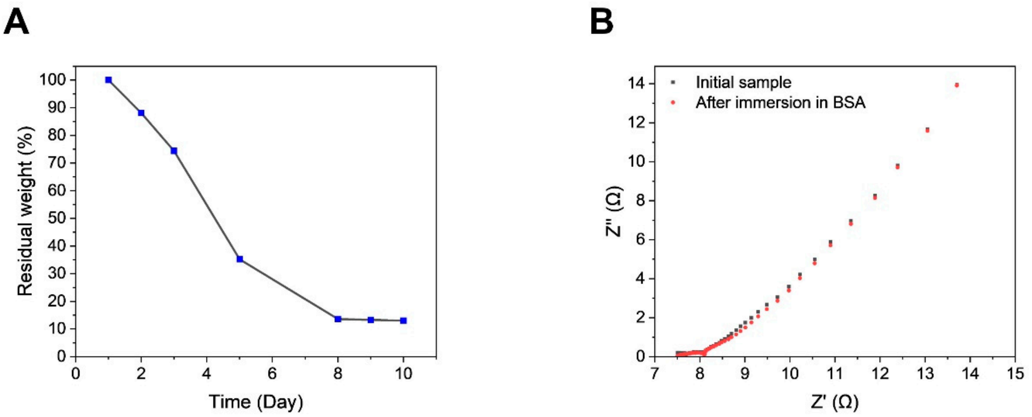

Figure S1 (A) Plot of the residual weight of the PEDOT−PVA/PEGDA DN hydrogels over time as it dries in air. (B) EIS plots of PEDOT−PVA/PEGDA DN hydrogels electrodes before and after being immersed in BSA solution for 24 h.

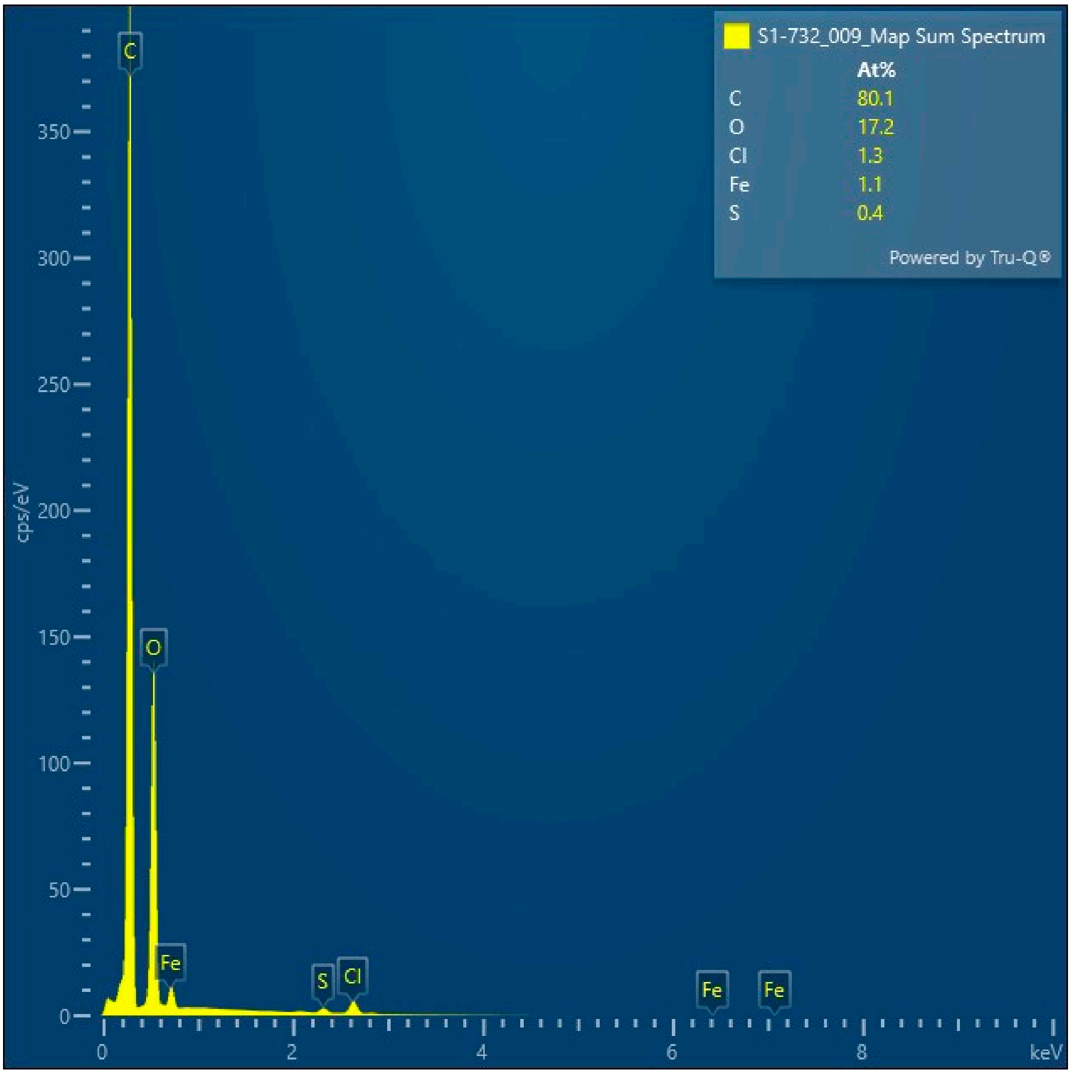

Figure S2 EDX mapping spectrum of the PEDOT−PVA/PEGDA DN hydrogel.

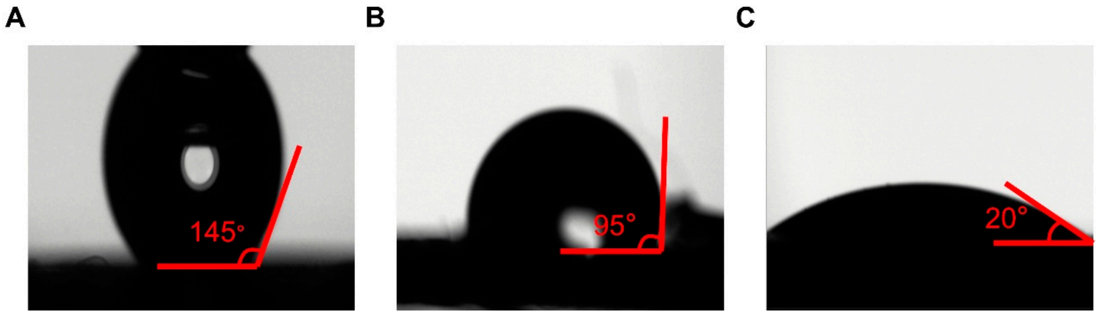

Figure S3 Contact angle profile of water on (A) an untreated bare carbon cloth surface and (B) a bare carbon cloth surface treated with HNO_3 (C) the PEDOT−PVA/PEGDA DN hydrogel surface.

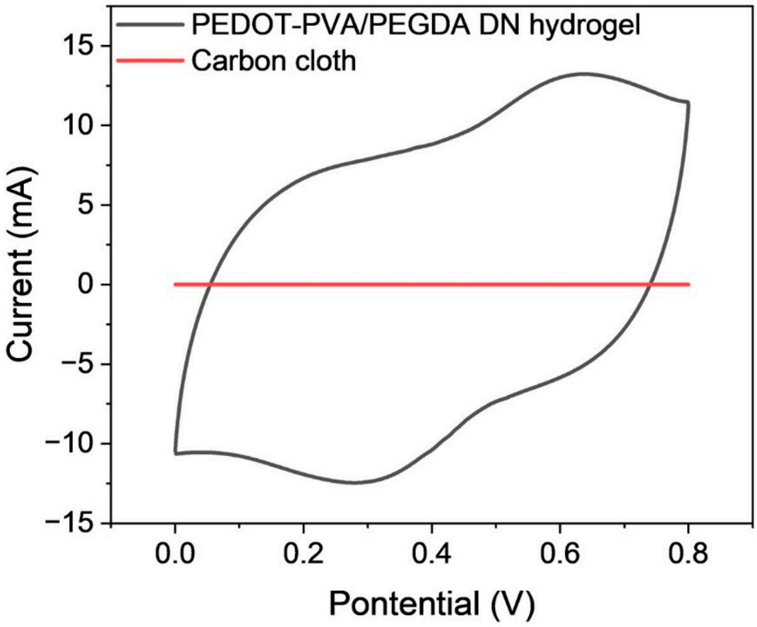

Figure S4 CV curves of PEDOT-PVA/PEGDA DN hydrogel electrode and bare carbon cloth.

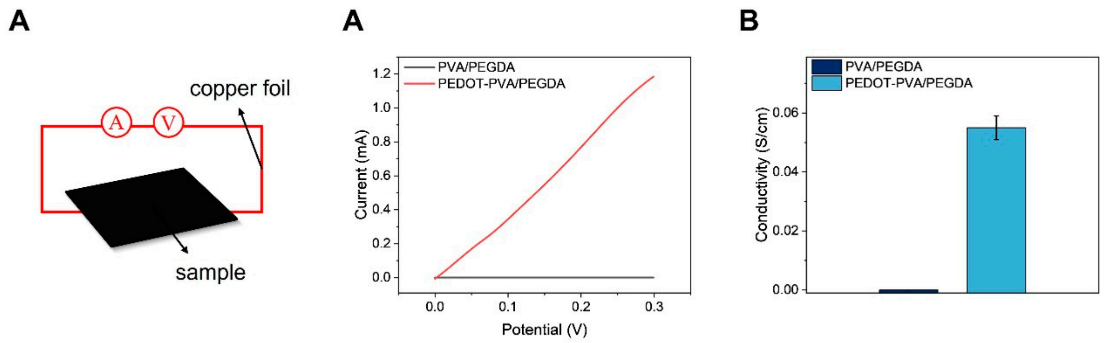

Figure S5 (A) Setup for conductivity measurement. The width, thickness and length of measured PEDOT−PVA/PEGDA DN hydrogels were 0.7 cm, 0.2 cm and 2 cm. (B) i-v curves of PVA/PEGDA DN hydrogel and PEDOT−PVA/PEGDA DN hydrogel. (C) Conductivity of the PVA/PEGDA DN hydrogel and PEDOT−PVA/PEGDA DN hydrogel.

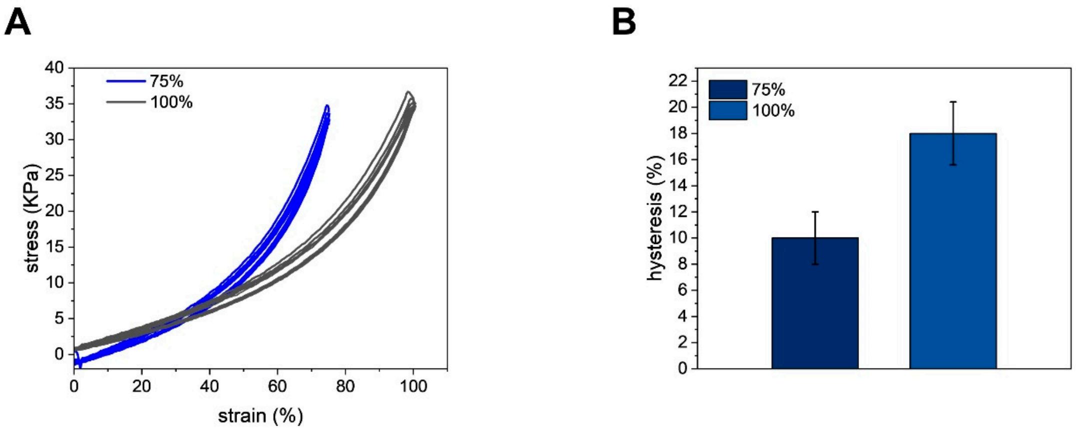

Figure S6 (A) Cyclic tensile test of PEDOT−PVA/PEGDA DN hydrogel with 75 % and 100% strain. (B) The tensile hysteresis values of the PEDOT-PVA/PEGDA DN hydrogel at 75%, 100% calculated from hysteresis loop.

Table S1 The parameters fitted from the Nyquist plot.

Element	Value	Error%
R_S	6.8	0.3
C	0.0002	11.9
R_{CT}	0.6	5.1
W	0.1	1.5
CPE	0.05	1.1

Table S2 The comparison of the PEDOT−PVA/PEGDA DN hydrogel-based supercapacitor with other PEDOT-based flexible supercapacitors.

PEDOT-based supercapacitor	Specific capacitance	Energy density	Power density	Stability	ref
PEGDA:PEDOT resin based 3D Printed Supercapacitor	19.5 mF cm^{-2} at 10 mV/s	0.7 uWh cm^{-2}	20 µW cm^{-2}	90% capacitance retention after 500 charge–discharge cycles	41
PEDOT: PSS/30 wt% V_2O_5 FSCs	14.8 at 5mV/s	1.37 uWh cm^{-2}	20 µW cm^{-2}	95.62% capacitance retention after 600 bending cycles at 90°	42
PEDOT:PSS/PVA/PMAA based SSC	7.4 mF cm^{-2} at 10 mV/s	0.65 uWh cm^{-2}	170 µW cm^{-2}	82% capacitance retention over 2000 charge-discharge cycles	43
PEDOT:PSS-LiTFSI-PVA based MSCs	44.5 F cm^{-2} at a discharge current of 0.1 mA cm^{-2}	4 uWh cm^{-2}	40 µW cm^{-2}	98% capacitance retention after 10000 charge–discharge cycles	44
CNT + PEDOT/PAAM/SA based SSC	128 mF cm^{-2} at 1 mA cm^{-2}	3.6 uWh cm^{-2}	200 µW cm^{-2}	96% capacitance retention after 5000 charge–discharge cycles.	45
PEDOT−PVA/PEGDA DN hydrogel-based supercapacitor	54.5 mF cm^{-2} at 10 mV s^{-1}	4.7 µWh cm^{-2}	213.9 µW cm^{-2}	97.6% capacitance retention after 3000 charge–discharge cycles	This work

Table S3 Composition of PEDOT−PVA/PEGDA DN hydrogels.

Sample No.	Sample Name	Network structure	PVA (w/w%)	PEGDA (w/w%)	EDOT (w/w%)
1	PVA/PEDOT	SN	10	0	10
2	PVA/PEGDA/ PEDOT	DN	10	10	10
3	PVA/PEGDA/ PEDOT	DN	10	20	10
4	PVA/PEGDA/ PEDOT	DN	10	30	10
5	PVA/PEGDA/ PEDOT	DN	10	40	10
6	PVA/PEGDA/ PEDOT	DN	10	50	10
7	PVA/PEGDA/ PEDOT	DN	10	30	13
8	PVA/PEGDA/ PEDOT	DN	10	30	18
9	PVA/PEGDA/ PEDOT	DN	10	30	23
11	PVA/PEGDA/ PEDOT	DN	10	30	25
12	PVA/PEGDA/ PEDOT	DN	10	30	28
